# Disentangling rhetoric and reality: an international Delphi study of factors and processes that facilitate the successful implementation of decisions to decommission healthcare services

**DOI:** 10.1186/s13012-014-0123-y

**Published:** 2014-09-10

**Authors:** Glenn Robert, Jenny Harlock, Iestyn Williams

**Affiliations:** National Nursing Research Unit, Florence Nightingale Faculty of Nursing & Midwifery, King’s College London, James Clerk Maxwell Building, 57 Waterloo Road, London, SE1 8WA England; Health Services Management Centre, School of Social Policy, Park House, University of Birmingham, Edgbaston, Birmingham, B15 2RT England

**Keywords:** Decommissioning, Delphi study, De-implementation, Disinvestment, Healthcare quality, Access to services, Rationing

## Abstract

**Background:**

The need to better understand processes of removing, reducing, or replacing healthcare services that are no longer deemed essential or effective is common across publicly funded healthcare systems. This paper explores expert international opinion regarding, first, the factors and processes that shape the successful implementation of decommissioning decisions and, second, consensus as to current best practice.

**Methods:**

A three round Delphi study of 30 international experts was undertaken. In round one, participants identified factors that shape the outcome of decommissioning processes; responses were analysed using conventional content analysis. In round two, responses to 88 Likert scale statements derived from round one were analysed using measures of the degree of consensus. In round three the statements that achieved low consensus were then repeated but presented alongside the overall results from round two. The responses were re-analysed to observe whether the degree of consensus had changed. Any open comments provided during the Delphi study were analysed thematically.

**Results:**

Participants strongly agreed that three considerations should ideally inform decommissioning decisions: quality and patient safety, clinical effectiveness and cost-effectiveness. Although there was less consensus as to which considerations informed such decisions in practice, those that drew the most agreement were: cost/budgetary pressures, government intervention and capital costs/condition. Important factors in shaping decommissioning were: strength of executive leadership, strength of clinical leadership, quality of communications, demonstrable benefits and clarity of rationale/case for change. Amongst the 19 best practice recommendations high consensus was achieved for: establishing a strong leadership team, engaging clinical leaders from an early stage, and establishing a clear rationale for change.

**Conclusions:**

There was a stark contrast between what experts thought should determine decommissioning decisions and what does so in practice; a contrast mirrored in the distinction the participants drew between the technical and political aspects of decommissioning processes. The best practice recommendations which we grouped into three categories—change management and implementation; evidence and information; and relationships and political dimensions—can be seen as contemporary responses or strategies to manage the tensions that emerged between the rhetoric and reality of implementing decommissioning decisions.

## Background

Healthcare systems have to balance the need to make efficiency savings whilst also maintaining high-quality care, and this creates challenges for those responsible for planning or commissioning services. As well as implementing new interventions and services, attention has increasingly turned to the need to cease performing activities that are no longer deemed essential or effective. A range of terms have been used to refer to such processes; for example, in this journal the term ‘de-implementation’ has been used to denote the abandonment of medical practices based on evidence relating to efficacy and harms [1].

The study presented in this paper employs the wider concept of decommissioning which encompasses de-implementation—replacement and removal as part of evidence-based practice at the organisational level—but also includes policies to remove interventions from across wider geographical areas and/or patient populations, and strategic reconfiguration of services leading to organisational downgrading or closure^a^. Illustrations of implementing decommissioning projects in England would include the following three examples.

- **Closure and relocation of local Walk-in-Centres**
  Following a local review of patient attendance at a local Accident and Emergency Department (A&E) and two Walk-in-Centres, it emerged there was duplication in the treatments being carried out for the same patient group with some treatments also being carried out at local exiting primary care practices. Early consultation with clinicians and staff revealed a majority decision to relocate one Walk in Centre to the A&E Department and close the remaining centre. Backlash from patients, the public, local elected politicians and also a small number of secondary care clinicians located at the A&E Department resulted in a review of the decision by the local Overview and Scrutiny Committee, and subsequently the Secretary of State for Health and national Independent Reconfiguration Panel. The decision was upheld. Continuous clinical backing and leadership were said to be key factors to successful implementation of the decision.
- **Attempt to remove and replace a drug from a formulary**
  Clinical sensory specialists are attempting to replace a drug for treatment for a sensory condition with an effective but less expensive alternative. Both drugs are manufactured by the same parent pharmaceutical company. The substitute drug is currently not licensed for treatment for the sensory condition and is not recommended by the National Institute for Clinical Excellence for that use in England. However early trials by clinicians have revealed similarly effective results, and the (unlicensed) drug is widely used internationally for the condition in private healthcare systems. Legal and licensing challenges, and the longevity of evidence trials, means that attempts to replace the drug have so far been unsuccessful. This has resulted in tensions for healthcare commissioners who perceive that the current treatment option consumes a disproportionate amount of resources.
- **Planned care home closures**
  A county council decided to reduce its reliance on care homes and thus embarked on a process of closing local care homes and relocating residents to neighbouring boroughs. A three-month consultation process with residents, relatives, and local community organisations took place before deciding to proceed with the programme of closures. Homes were closed one by one over a period of four years to allow the market to respond to the demand for alternative beds. Each home took approximately nine months to close. An experienced officer was designated to project manage each closure and liaise with residents, relatives, as well as care staff and medical professionals supporting residents to ensure a smooth transition. Planned timescales, communication with residents and their families, and engaging the whole support circle of care staff and medical professionals were key factors to success.

Other types of decommissioning would include closure of an acute service and its reopening in a community setting, nationwide reconfiguration of (for example) children’s healthcare services, and the de-commissioning of obsolete medical technologies.

Despite examples of what might be viewed as successful decommissioning projects, there is a growing consensus that such decommissioning is something of an Achilles’ heel for healthcare systems [2,3]. The published literature in this area also lacks a rigorous analysis of the contextual factors that facilitate or hinder practice [4]. Efforts have been made to generate lists of candidates for decommissioning [5,6]. These are invariably established following application of quality and cost-effectiveness calculations relative to a comparator. However, the perils of decision implementation remain largely unexplored and unexplained in these accounts; in the absence of such guidance, the danger is that blunt and unsophisticated decommissioning approaches are employed, leading to unnecessary turmoil in their implementation and with little guarantee of positive outcomes [7], and/or unanticipated delays and costs.

Developing better understanding of how decommissioning programmes unfold is therefore a crucial first step towards providing evidence-based guidance for future policy and practice. This forms the overall aim of the study reported in this paper which addresses two key research questions: first, what is international expert opinion regarding best practice in decommissioning healthcare services? Second, what factors and processes facilitate the successful implementation of decisions to decommission healthcare services? Below we present our methods and findings before discussing the implications for future decommissioning research and practice.

## Methods

Given the lack of published evidence as to ‘what works’ in terms of decommissioning we undertook a Delphi study of international experts as an economical and as objective as possible way of exploring current consensus relating to this complex policy challenge. Delphi studies build consensus by collecting data from a panel of experts through iterative questionnaires and are effective in establishing consensus in complex topic areas [8,9]. The Delphi method was developed by the RAND Corporation in the 1950s and was originally used to forecast the emergence of new technologies [10]. A similar method has been used in more recent decades to establish research priorities in several areas of healthcare [11-16], as well as to generate consensus on policy issues [17-19].

A Delphi study exploring consensus in a policy area is a variant on the original approach but has the same typical features: an expert panel; a series of rounds in which information is collected from participants, analysed and fed back as the basis for subsequent rounds; at least one opportunity for participants to revise their judgments on the basis of the group feedback; and anonymity for the participants. With regard to this latter feature, participants in Delphi studies never meet or interact directly but are sent questionnaires and asked to record their views. Often they are asked initially to suggest the factors or cues that should be considered by the group. Having contributed to drawing up the agenda, the participants are then sent a questionnaire that seeks their individual views on the items that they and their co-participants have suggested. The responses are collated by the organisers and returned to the participants in summary form, usually indicating the group judgment and the individual’s initial judgment. Participants are given the opportunity to revise their judgment in the light of the group feedback. The process may be repeated a number of times before the judgments of the participants are statistically aggregated, sometimes after weighting for expertise [15].

In this study, three key stakeholder groups—each comprising approximately 10 people—were purposively selected for their expertise in researching, leading or implementing decommissioning programmes. The three groups were: academics/researchers; policy-makers and regulators, and; commissioners and providers of healthcare services (Table 1).

**Table 1:** **Participants in Delphi study**

	**No. participants**
**Country:**	
Australia	6
Canada	3
United Kingdom	20
Ireland	1
**Perspective:**	
Academic	8
Policy maker	10
Practitioner	12
**Expertise in forms of decommissioning:**	
Removal or replacement of a treatment from a formulary or patient pathway	2
Relocation/replacement of a service as part of reconfiguration	5
Relocation/replacement of a service from an acute to a community setting	1
Closure or partial closure of a service	2
Closure or partial closure of an acute healthcare organisation	1
More than one of above (and including ‘research/policy development’)	19

Expertise was conferred by extent of individual experience of decommissioning (either in research or practice settings) or knowledge of and/or responsibility for decommissioning policy within a healthcare system. Candidates for the ‘research’ group were identified through a review of the published literature^a^. Candidates for the ‘policy’ and ‘practice’ groups were identified through desktop searches and nominations from an international advisory group for the research project. These activities resulted in a list of approximately 100 potential respondents from the United Kingdom (UK), Europe, North America, Australia and New Zealand, all of who were invited to participate via an email from the research team. Of these, 30 agreed to participate. As Table 1 shows, the final participants were drawn from the UK, Australia, Canada and the Republic of Ireland but it should be noted that the final sample of participants, although international, contains a relative over-representation of UK respondents, especially in the ‘practice’ category.

Each participant was asked to consider, define, and rate criteria and factors that shape the process and outcomes of decommissioning programmes by means of three iterative rounds. They were asked to complete each online round within one week and to provide examples of ‘best practice’ in decommissioning throughout the process. Analysis of all open comment responses was undertaken by one researcher (GR) within and—at the end of the Delphi study—across the three rounds; all open comments were extracted from each response and analysed using open coding and constant comparison. Similar codes were then grouped to identify key concepts emerging from the data. Consensus was statistically operationalised by measuring whether the group’s ratings were strongly polarized (*e.g.*, 50% respondents strongly agreeing and 50% strongly disagreeing with any statement is a strongly polarized distribution).

In round one, participants were asked to nominate up to five:

1. Considerations that should inform decisions to implement decommissioning.
2. Considerations that do inform decisions to implement decommissioning.
3. Factors that positively shape the process of decommissioning.
4. Factors that negatively shape the process of decommissioning.
5. Factors that positively shape the outcome of decommissioning.
6. Factors that negatively shape the outcome of decommissioning.

They were also invited to make best practice recommendations with regards to implementing decommissioning decisions. Open-comment fields allowed participants to provide explanations of and justification for their suggestions, and to raise any questions or issues relating to the study. The anonymised, cumulative responses were then fed back to the whole panel to inform round two.

In round two, participants were asked to rank their level of agreement with statements derived from round one, using a four point Likert rating scale (strongly disagree, disagree, agree, and strongly agree). Participants were then asked to rank the relative importance in shaping the process and outcomes of decommissioning of the factors put forward in round one, again using a four point Likert rating scale (very low importance, little importance, high importance, very high importance). Open comment fields allowed participants to explain their responses. The level of consensus achieved in relation to the total of 88 rating scale questions included in round two was assessed using the following thresholds for level of consensus [20]:

1. high = 70% of ratings in one category or 80% in two contiguous categories
2. medium = 60% of ratings in one category or 70% in two contiguous categories
3. low = 50% of ratings in one category or 60% in two contiguous categories
4. none ≤60% in two contiguous categories

The anonymised, cumulative responses relating to those factors that achieved low or no consensus were then fed back to the whole Delphi panel to inform round three.

In round three, participants were asked to reflect and comment on the round two results for the 17 statements that achieved low or no consensus, and rank their agreement with the 17 statements having had an opportunity to review the results from the panel as a whole. The final outcomes from rounds one to three were fed back to all participants and further open comments invited. Ethics approval was granted by the University of Birmingham Humanities and Social Sciences Ethical Review Committee, reference number ERN_13-0172.

## Results

The results of each of the three rounds of the Delphi study are presented in turn below. These are presented together with illustrative quotations of the main findings from the analysis of the open comments provided by respondents in each round. The brackets after each quotation indicate the country of work of the respondent and whether their expertise relates to research, policy, or practice in decommissioning processes.

### Round one

In round one, the 30 individual respondents put forward a total of 142 criteria that they believed should inform decisions to carry out decommissioning, and 126 criteria that they believed did inform such decisions in practice. As Table 2 shows, after combining and aggregating similar suggestions, sixteen common criteria were identified as informing decisions to carry out decommissioning both as it should be done and is done in practice. In addition, a further eight considerations were identified as informing decisions in practice.

**Table 2:** **Round one aggregated responses from 30 participants: considerations that (a) should and (b) do in practice inform decommissioning decisions (listed in alphabetical order)**

**Common considerations that both (a) should, and (b) do in practice inform decisions:**	
1	Addressing inequalities
2	Alignment with strategic priorities
3	Availability of alternative services/interventions
4	Capital costs/condition (buildings, maintenance)
5	Clinical effectiveness
6	Cost/budgetary pressures
7	Cost-effectiveness/efficient use of resources
8	Cost of implementation of decommissioning
9	Duplication of services
10	Equitable resource allocation
11	Evidence-base
12	Maximizing population health
13	New service developments/innovations
14	Patient and public views
15	Quality and patient safety
16	Responding to changing demographics/population needs
**Additional considerations in practice:**	
1	Complexity of implementation of decommissioning
2	Government intervention, *e.g.*, legislation
3	Impact on workforce
4	Marginal groups not heard (*e.g.*, homeless)
5	Prejudice against public sector provision
6	Support from clinicians
7	Support from elected politicians
8	Support from industry and other interest groups

Open comments provided by participants to explain or support their suggestions included that, in practice, decommissioning decisions are not necessarily always ‘evidence-based’ (a common theme throughout all three rounds of the Delphi) and, rather, are often driven by a financial (cost-saving) imperative:

‘[We] need to acknowledge that health services are delivered within a dynamic policy and political context that on occasions drive decommissioning contrary to evidence’. (Australia, policy/practice)

‘…Whilst the ideal is decommissioning of services that don’t represent high quality care or value for money, the reality is that decommissioning decisions are generally made as a money saving mechanism’. (UK, practice)

‘Cutting costs is a legitimate reason for change but it is often hidden from public view or presented within the case for change as a subsidiary factor’. (UK, policy)

Individual respondents also identified a total of 463 factors that positively or negatively shape the process or outcomes of decommissioning. These were combined and aggregated into the 30 factors listed in Table 3 and each placed in one of three broad categories that emerged as we reviewed the factors: change management and implementation strategy; evidence and information; and relationships and political dimensions. The large number of factors identified by participants defied simple categorisation. However, for the pragmatic purpose of presenting the findings from round one back to participants in a clear and structured way—and to aid our overall analysis—we grouped the factors into these three broad themes (whilst recognising that they are to some extent related and not mutually exclusive).

**Table 3:** **Round one aggregated responses from 30 participants: factors that positively or negatively shape the process or outcomes of decommissioning (listed in alphabetical order)**

**Change management and implementation strategy:**	
1	Attention throughout to human aspects of process of change
2	Availability of resources to support decision-making and implementation processes
3	Clarity of incentives and levers to support change
4	Clarity of specific aims and objectives at start
5	Extent of cultural and behavioural change
6	Pace of change
7	Quality of strategic planning
8	Quality of project management
9	Complexity of decommissioning programme
10	Quality of communication
11	Strength of clinical leadership
12	Strength of executive leadership
13	Training and preparedness of staff
**Evidence and information:**	
14	Availability of alternative services
15	Clarity around new patient pathways
16	Clarity of evidence/data to support business case, ongoing monitoring and impact assessment
17	Demonstrable benefits
18	Extent of adoption elsewhere of new intervention/service
19	Review/evaluation of process
**Relationships and political dimensions:**	
20	Clarity of rationale/case for change
21	Extent to which challenges vested interests
22	Level of political support
23	Meets community expectations
24	Nature and extent of clinician engagement/involvement
25	Nature and extent of media coverage
26	Nature and extent of patient/public involvement
27	Quality of partnership working with relevant agencies
28	Reputation of existing providers
29	Stability within the local health economy during transition
30	Transparency of decision-making process

Participants provided explanations for their suggestions in their open comments as illustrated below:

‘Effective leadership that can navigate the political aspects associated with decommissioning is key. This needs leadership at various levels and make sure the right people lead and engage with the different stakeholder groups—so the medical profession is key to the process—examples include having a doctor to speak to the general public’. (Australia, research)

‘Decommissioning healthcare services is difficult unless you have clinicians onside. This also needs to be complemented by political support at the local and national level. Without these and a clear narrative of what these changes are and why these are necessary then decommissioning is difficult to bring about’. (Australia, research)

‘Lots of decommissioning I see seems to focus on a technical, rational decision to change services—but doesn’t pay sufficient attention to the politics of such change (at its peril)’. (UK, policy)

‘A very rigid ‘science of healthcare’ and therefore what is worth commissioning is causing the decommissioning of ‘non evidence-based’ services in a way that appears fair but is hugely damaging. The evidence-base used is flawed’. (UK, practice)

Finally in round one, individual respondents proposed a total of discrete 125 ‘best practice’ recommendations relating to implementing decommissioning which were combined and aggregated into 19 ‘best practice’ recommendations by the authors (Table 4).

**Table 4:** **Round one aggregated responses from 30 participants: best practice recommendations (listed in alphabetical order)**

1	Adopt a whole systems perspective from the beginning
2	Base decisions on evidence of what works
3	Be pro-active in engaging with the media
4	Clear and thorough project planning and governance
5	Collect and analyse relevant data before, during and after
6	Do not decommission until alternative services in place
7	Engage and involve clinical leaders from an early stage
8	Engage and involve service users from an early stage
9	Ensure a transparent decision-making process
10	Establish a clear rationale and narrative for change
11	Establish clear criteria by which to measure outcomes
12	Focus on improved patient experience and quality
13	Identify and establish a strong top leadership team
14	Pay attention to the human elements of change and the impact that decommissioning can have on those involved
15	Pay equal attention to implementation and decision-making phases
16	Place emphasis on public engagement and communication
17	Provide regular feedback on progress
18	Resource the process and retain a budget for contingencies
19	Secure high-level political support (national and local) at early stage

Again, participant comments provided further justification for their suggested recommendations, often drawing attention to the ‘political’ nature of decommissioning processes:

‘Once a decision has been taken it should be stuck to as long as the appropriate process has been followed. Many decommissioning projects fail to achieve what they set out to achieve because commissioners and organizations go back on their decisions. Amongst the main reasons why commissioners may change their minds are the influences of staff groups who may not have been fully engaged in the decision-making process and politicians who may have been influenced by public opinion to oppose change’. (UK, research)

‘Decommissioning services is a taboo subject; in order to manage it effectively you need a good strong clinical leader so that the service is decommissioned from a clinical perspective on quality issues and ineffectual clinical outcomes’. (UK, practice)

### Round two

Twenty-seven of the 30 respondents in round one participated in the round two survey which assessed the extent to which there was consensus amongst the respondents as to which of the considerations identified in round one (see Table 2) *should* inform decisions to carry out decommissioning, and which do inform decisions in practice. Tables 5 and 6 present these results.

**Table 5:** **Round two results: to what extent do you agree that the following considerations should ideally inform decisions to carry out decommissioning?**

**Consideration**	**Strongly disagree**	**Disagree**	**Agree**	**Strongly agree**	**Do not know**
Quality and patient safety	0	0	7.4	92.6	0
Clinical effectiveness	0	0	11.1	88.9	0
Cost-effectiveness/efficient use of resources	0	0	18.5	81.5	0
Duplication of services	0	0	33.3	66.7	0
Evidence-base	0	3.7	29.6	63.0	3.7
Responding to changing demographics/population needs	0	0	48.1	48.1	3.7
Addressing inequalities	0	7.7	30.8	61.5	0
Maximising population health	0	7.4	37.0	55.6	0
Alignment with strategic priorities	0	11.1	40.7	48.1	0
New service developments/innovations	0	0	66.7	29.6	3.7
Equitable resource allocation	3.7	7.4	48.1	40.7	0
Patient and public views	0	18.5	40.7	40.7	0
Cost/budgetary pressures	0	18.5	48.1	33.3	0
Availability of alternative services/interventions	0	14.8	63.0	22.2	0
Cost of implementation of decommissioning	14.8	11.1	44.4	25.9	3.7

(% responses) (in descending order of average strength of agreement).

**Table 6:** **Round two results: to what extent do you agree that the following considerations do actually—in practice—inform decisions to carry out decommissioning?**

**Consideration**	**Strongly disagree**	**Disagree**	**Agree**	**Strongly agree**	**Do not know**
Cost/budgetary pressures	3.7	0	22.2	74.1	0
Government intervention (*e.g.*, legislation)	7.4	3.7	37.0	51.9	0
Capital costs/condition (buildings, maintenance)	3.8	15.4	42.3	30.8	7.7
Quality and patient safety	0	7.4	66.7	25.9	0
Complexity of implementation of decommissioning	0	29.6	40.7	18.5	11.1
Support from clinicians	7.4	18.5	37.0	33.3	3.7
Cost-effectiveness/efficient use of resources	3.7	22.2	48.1	25.9	0
Support from industry and other interest groups	7.4	29.6	37.0	14.8	11.1
Clinical effectiveness	3.7	11.1	74.1	11.1	0
Availability of alternative services/interventions	3.7	29.6	44.4	18.5	3.7
Cost of implementation of decommissioning	0	33.3	48.1	14.8	3.7
Alignment with strategic priorities	7.4	29.6	37.0	22.2	3.7
Support from elected politicians	11.1	14.8	51.9	22.2	0
New service developments/innovations	8.0	28.0	44.0	16.0	4.0
Prejudice against public sector provision	3.7	51.9	22.2	7.4	14.8
Duplication of services	7.4	33.3	40.7	14.8	3.7
Responding to changing demographics/population needs	7.4	37.0	40.7	11.1	3.7
Evidence-base	3.7	37.0	51.9	7.4	0
Impact on workforce	3.7	59.3	18.5	14.8	3.7
Patient and public views	11.1	40.7	37.0	7.4	3.7
Maximising population health	11.1	48.1	37.0	0	3.7
Addressing inequalities	11.1	70.4	14.8	3.7	0
Equitable resource allocation	11.1	74.1	11.1	3.7	0
Marginal groups not heard (*e.g.*, homeless)	22.2	66.7	7.4	0	3.7

(% responses) (in descending order of average strength of agreement).

Respondents were also asked to rank the relative importance of the factors proposed in round one as shaping—either positively or negatively—the process or outcomes of decommissioning. The results are shown in Table 7.

**Table 7:** **Round two results: rating of factors in terms of importance within each category in shaping the extent to which decommissioning is implemented as planned**

**Factor**	**Very low importance**	**Little importance**	**High importance**	**Very high importance**	**Do not know**
**Change management and implementation strategy**					
Strength of executive leadership	0	0	7.4	92.6	0
Strength of clinical leadership	0	3.7	14.8	74.1	7.4
Quality of communication	0	3.7	33.3	63.0	0
Clarity of specific aims and objectives at start	0	3.7	48.1	48.1	0
Extent of cultural and behavioural change	0	7.4	51.9	40.7	0
Attention throughout to human aspects of process of change	0	11.5	46.2	42.3	0
Quality of project management	0	11.1	48.1	40.7	0
Availability of resources to support decision-making and implementation processes	0	14.8	48.1	33.3	3.7
Quality of strategic planning	0	11.1	63.0	25.9	0
Training and preparation of staff	0	18.5	63.0	18.5	0
Clarity of incentives and levers to support change	0	22.2	59.3	18.5	0
Complexity of decommissioning programme	0	29.6	59.3	11.1	0
Pace of change	3.7	40.7	51.9	0	3.7
**Evidence and information**					
Demonstrable benefits	0	0	37.0	63.0	0
Clarity of evidence/data to support business case, ongoing monitoring and impact assessment	0	0	63.0	37.0	0
Clarity around new patient pathways	0	14.8	63.0	18.5	3.7
Review/evaluation of process	3.7	11.1	59.3	25.9	0
Availability of alternative services	0	25.9	51.9	22.2	0
Extent of adoption elsewhere of new intervention/service	0	33.3	48.1	18.5	0
**Relationships and political dimensions**					
Clarity of rationale/case for change	0	0	29.6	70.4	0
Nature and extent of clinician engagement/involvement	0	7.4	33.3	59.3	0
Level of political support	0	14.8	29.6	55.6	0
Transparency of decision-making process	0	7.4	63.0	29.6	0
Nature and extent of patient/public engagement/involvement	0	14.8	51.9	29.6	3.7
Quality of partnership working with relevant agencies	0	7.7	76.9	11.5	3.8
Extent to which challenges vested interests	0	23.1	46.2	30.8	0
Nature and extent of media coverage	0	25.9	51.9	18.5	3.7
Stability within the local health economy during transition	3.7	18.5	66.7	11.1	0
Reputation of existing providers	0	33.3	55.6	3.7	7.4
Meets community expectations	0	37.0	51.9	7.4	3.7

(% responses) (in descending order of importance within each category).

The political and contested nature of decommissioning decisions and the key role of clinical support in implementing such decisions were again highlighted in participants open comments:

‘Politicians may not instigate decommissioning decisions themselves but they can bully senior decision makers into changing their plans if they feel that they will adversely effect their election chances. Other decommissioning decisions are taken at short notice and they need to deliver savings quickly and effectively. If a decommissioning decision costs money in the short term or is hard to implement and will take too long then it won’t happen’. (UK, research)

‘Clinical support for decommissioning can make or break a project’. (UK, research)

‘Decommissioning projects fail when the clinical case is not clear or supported by clinicians and the public’. (UK, policy)

Analysis of the degree of consensus in the groups response to the 88 rating scale questions included in round two found that: 59 questions achieved a ‘high’ consensus amongst participants (with very strong consensus around 12 questions in particular); 11 achieved ‘medium’ consensus; 10 achieved ‘low consensus’; and 8 achieved ‘no consensus’. The nine statements with very high consensus amongst respondents were:

1. ‘Cost-effectiveness/efficient use of resources’ considerations should ideally inform decisions to carry out decommissioning (strongly agree)
2. ’Quality and patient safety’ considerations should ideally inform decisions to carry out decommissioning (strongly agree)
3. ‘Clinical effectiveness’ considerations should ideally inform decisions to carry out decommissioning (strongly agree)
4. ‘Cost/budgetary pressures’ considerations do actually—in practice—inform decisions to carry out decommissioning (strongly agree)
5. ‘Addressing inequalities’ considerations do actually—in practice—inform decisions to carry out decommissioning (disagree)
6. ‘Strength of executive leadership’ has very high importance for shaping the extent to which decommissioning is implemented as planned
7. ‘Strength of clinical leadership’ has very high importance for shaping the extent to which decommissioning is implemented as planned
8. ‘Clarity of rationale/case for change’ has very high importance for shaping the extent to which decommissioning is implemented as planned
9. ‘Quality of partnership working with relevant agencies’ has high importance for shaping the extent to which decommissioning is implemented as planned

The three ‘best practice recommendations’ with very high consensus amongst respondents were:

1. Identify and establish a strong top leadership team (very high importance)
2. Establish a clear rationale and narrative for change (very high importance)
3. Engage and involve clinical leaders from an early stage

Fourteen of the 18 questions with relatively little (or no) consensus related to the extent to which participants agreed that nominated factors do inform decisions to carry out decommissioning in practice. The remaining five sections in round two saw much greater overall levels of consensus. For example, all of the questions in the section exploring the extent to which the nominated factors should inform decisions to carry out decommissioning achieved a ‘medium’ or ‘high’ consensus.

The following seven best practice recommendations were identified as being the most important (in descending order of importance):

1. Identify and establish a strong leadership team.
2. Engage and involve clinical leaders from an early stage.
3. Establish a clear rationale and narrative for change.
4. Clear and thorough project planning and governance.
5. Secure high level political support (national and local) at an early stage.
6. Base decisions on evidence of what works.
7. Adopt a whole systems perspective from the beginning.

### Round three

Given the stark contrast in expert consensus between what should happen and what happens in practice in relation to decommissioning decisions (one of the participants reflected that ‘the differences between ‘should’ and ‘do’ are real’), we explored this theme further in the third and final round. We asked participants to reconsider the 18 questions in round two that registered little or no group consensus; 13 of these related to the ‘what happens in practice’ section. Twenty five of the 27 respondents in round two participated in the round three survey and Table 8 presents the results.

**Table 8:** **Comparisons of round two and round three responses for statements attracting no or low consensus in round 2**

	**Strongly disagree**		**Disagree**		**Agree**		**Strongly agree**		**Do not know**		**Change in level of consensus**
	**Round 2**	**Round 3**	**Round 2**	**Round 3**	**Round 2**	**Round 3**	**Round 2**	**Round 3**	**Round 2**	**Round 3**	
‘Costs of implementation of decommissioning’ do actually in practice inform decision to carry out decommissioning	0	0	33.3	32.0	48.7	60.0	14.8	8.0	3.7	0	low → medium
‘Duplication of services’ does actually in practice inform decision to carry out decommissioning	7.4	0	33.3	24.0	40.7	68.0	14.8	8.0	3.7	0	no → medium
The ‘evidence base’ does actually in practice inform decision to carry out decommissioning	3.7	0	37.0	32.0	51.9	52.0	7.4	16.0	0	0	no change (low)
‘Maximising population health’ does actually in practice inform decisions to carry out decommissioning	11.1	4.0	48.1	76.0	37.0	20.0	0	0	3.7	0	no → high
‘Availability of alternative services/interventions’ does actually in practice inform decisions to carry out decommissioning?	3.7	0	29.6	16.0	44.4	68.0	18.5	12.0	3.7	4.0	low → medium
‘Responding to changing demographics/population needs’ does actually in practice inform decisions to carry out decommissioning	7.4	0	37.0	44.0	40.7	48.0	11.1	4.0	3.7	4.0	no change (none)
‘New service developments/innovations’ do actually in practice inform decisions to carry out decommissioning?	8.0	0.0	28.0	24.0	44.0	72.0	16.0	4.0	4.0	0	low → high
‘Alignment with strategic priorities’ does actually in practice inform decisions to carry out decommissioning	7.4	0	29.6	28.0	37.0	64.0	22.2	8.0	3.7	0	no → medium
‘Patient and public views’ do actually in practice inform decisions to carry out decommissioning	11.1	8.0	40.7	72.0	37.0	16.0	7.4	4.0	3.7	0	no → high
‘Support from industry and other interest groups’ do actually in practice inform decisions to carry out decommissioning	7.4	0	29.6	32.0	37.0	48.0	14.8	4.0	3.7	16.0	no change (none)
‘Prejudice against public sector provision’ does actually in practice inform decisions to carry out decommissioning	3.7	8.0	51.9	60.0	22.2	8.0	7.4	8.0	14.8	16.0	low → medium
‘Complexity of implementing decommissioning’ does actually in practice inform decisions to carry out decommissioning	0	0	29.6	8.0	40.7	72.0	18.5	16.0	11.1	4.0	no → high
‘Impact on workforce’ does actually in practice inform decisions to carry out decommissioning	3.7	8.0	59.3	48.0	18.5	28.0	14.8	12.0	3.7	4.0	low → no
	**Very little importance**		**Little importance**		**High importance**		**Very high importance**		**Do not know**		**Change in level of consensus**
	**Round 2**	**Round 3**	**Round 2**	**Round 3**	**Round 2**	**Round 3**	**Round 2**	**Round 3**	**Round 2**	**Round 3**	
What is the relative importance of the ‘pace of change’ for shaping the extent to which decommissioning is implemented as planned?	3.7	0	40.7	33.3	51.9	50.0	0.0	4.2	3.7	12.5	no change (none)
What is the relative importance of the ‘extent of adoption elsewhere of new intervention/service’ for shaping the extent to which decommissioning is implemented as planned?	0	0	33.3	24.0	48.1	60.0	18.5	8.0	0	8.0	low → medium
What is the relative importance of ‘meets community expectations’ for shaping the extent to which decommissioning is implemented as planned?	0	0	37.0	28.0	51.9	64.0	7.4	4.0	3.7	4.0	low → medium
What is the relative importance of the ‘reputation of existing providers’ for shaping the extent to which decommissioning is implemented as planned?	0	0	33.3	12.0	55.6	76.0	3.7	4.0	7.4	8.0	low → high

Participants also commented on why they felt these particular questions had achieved no or low consensus in round two. For example, in relation to whether the costs of decommissioning do in practice inform decisions, participants reflected that:

‘I can remember four years of meetings with officers from a handful of other bodies all relating to a decommissioning which was mainly intended to secure a service improvement, rather than a cash saving. The time we spent on the exercise was never counted as a cost … ’. (UK, practice)

‘There is a major misconception of cost. The costs incurred are far more complex than I believe is considered … ’. (UK, practice)

‘i don’t really think the cost per se are relevant—it is more about skills, having the right framework in place, leadership and political will … the failure to see priority setting as a programme … which needs resources attached to it is one of the reasons we have poor priority setting … it is a lack of understanding and will to establish priority setting as a major programme needing a team and resources to support it’. (UK, policy/practice)

At the end of the third round of the Delphi study no or low consensus remained amongst the experts in relation to five of the original 88 statements generated in round one. These were:

1. ‘The ‘evidence base’ does inform decisions to carry out decommissioning.
2. ‘Responding to changing demographics/population needs’ does inform decisions to carry out decommissioning.
3. ‘Support from industry and other interest groups’ does inform decisions to carry out decommissioning.
4. ‘Impact on workforce’ does inform decisions to carry out decommissioning.
5. What is the relative importance of the ‘pace of change’ for shaping the extent to which decommissioning is implemented as planned?

With regard to the first of these—the extent to which participants agreed that the ‘evidence base’ does actually in practice inform decisions to carry out decommissioning—the open comments provided by participants revealed the widest range of reactions to the round two rankings:

‘Very disconcerting ‘disagree’ response rate. Evidence certainly ought to inform decisions and in my applied experience it is central. Please unpack and report the reasons for the ‘disagree’ responses’. (Australia, research)

‘I think we have all experienced decisions that were taken in the absence of a robust evidence base. I don’t think I have personally experienced decisions that were taken in flat contradiction of a robust evidence base. For that reason I choose ‘agree’ but recognize others may choose ‘disagree’’. (UK, practice)

‘I’m afraid I just don’t buy the standard text book answer here—it feels more an issue of responding to national/local politics and a pragmatic sense of what we can get away with in practice’. (UK, policy)

‘In my experience, I still feel that a lot of decommissioning decisions are taken on ‘gut feeling’ rather than necessarily on the basis of hard evidence or facts’. (UK, research)

‘my experience is that a highly reductionist approach to evidence-base and efficacy tends to negate what actually works/the effectiveness in real life … services that work on paper often don’t work in practice. The biggest block to truly evidence-based decommissioning is a lack of effectiveness data and a lack of review of the beliefs around efficacy’. (UK, practice)

We return to the question of the role of evidence in decommissioning in our discussion below.

## Discussion

We set out to establish through a three round Delphi study, firstly, the state of current international opinion regarding best practice in decommissioning healthcare services. Our expert participants were able to generate a list of recommendations to this end which we grouped under three categories: change management and implementation; evidence and information; and relationships and political dimensions. We also sought to explore what factors and processes facilitate the successful implementation of decisions to decommission healthcare services. Here we received a wide range of responses and in the preceding results section we detail the strength and direction of consensus with regard to these.

One inference that could be drawn from our overall results is an apparent distinction between ‘good’ and ‘bad’ rationales for implementing a decommissioning decision; the former relating to a desire to improve healthcare by withdrawing an ineffective treatment or service and the latter relating to decisions that are driven purely by a financial (*i.e.*, cost-saving) motive. In practice, decommissioning does not, although in some cases it may, necessarily mean just the complete withdrawal of a service but rather its replacement with something more cost-effective. In other words the financial ‘motive’ is not, necessarily, a ‘bad’ thing. However, we would argue that in the current context of austerity such a desired scenario can more often be distorted. We would therefore argue that cost is not necessarily—in itself—an illegitimate reason to decommission; rather it is the tendency to adopt a short-term perspective whilst searching for a ‘quick fix’—instead of taking a whole systems perspective based on considerations of longer-term sustainability—that is problematic.

We now turn to interpreting our results in terms of the current state of the science (and art) of decommissioning, and by using the three categories that emerged from our analysis of the round one responses and were key overarching thematic categories for the remainder of the Delphi study.

### Change management and implementation

Notwithstanding the apparent departure from rational models of decision-making propounded in much of the extant literature (and echoed in our respondents views on what should drive decommissioning), there did nonetheless appear to be some clear guiding recommendations emerging from the Delphi study for helping those implementing decommissioning processes to navigate what the participants clearly regarded as a highly political and contested landscape. These were located principally on the change process, and the role of negotiation, sense-making and leadership in achieving this. We were struck by the extent of overlap between these recommendations and those contained in mainstream change management thinking. For example, one participant commented that the list of best practice recommendations is: ‘… all about crafting a narrative, paying attention to the politics and bringing people with you—more about the skills of managing change than about the technicalities of the decision taken’ (UK, policy, round three).

### Evidence and information

Two of the open comments made in the course of the Delphi study resonated very strongly with the general sense of responses overall; they were that decommissioning is ‘highly subjective and not based on objective evidence’ (UK, practice, round three) and that it ‘isn’t about traditional rational models of evidence-based policy and practice—but a more political and messy process of ‘muddling through as best we can’ (UK, policy, round three). Despite this, the nature of the ‘evidence’ commonly used to underpin decommissioning decisions does appear to be consistent in one important respect; a common perception in our findings was that it is not patient or service user views but rather cost data from provider organizations that is prioritized when ‘building a case’ and implementing decommissioning decisions. The general lack of an evidence-based approach to decommissioning in practice is likely influenced by many factors including the burden associated with collecting and analysing longitudinal data as well as the recognised challenges not only of undertaking ‘gold standard’ evaluation methods—like randomized controlled trials—in the case of often complex interventions but also translating the resulting findings into practice. More specific to decommissioning is the challenge of collecting data to disprove the worth of a service or intervention especially in the current competitive funding environment which only serves to increasingly challenge healthcare providers to ‘prove’ the beneficial impact of their services. More broadly, Greenhalgh *et al.* [21] recently provided a trenchant critique of what they perceive as a ‘crisis’ in the evidence-based medicine movement. Several of the limitations they identify resonate strongly with what appears to be the contemporary approach to decommissioning decisions. These include the implementation of policies based on political conviction (rather than a robust and rigorous evidential base), that the ‘sheer volume’ of evidence has become unmanageable (we would argue for policy makers/managers as well as for individual clinicians), ‘scant attention [being paid] to opportunity costs or unintended human and financial consequences’ , and the crowding out of ‘local, individualised’ patient centred care.

The overall ‘messiness’ of decommissioning processes was a recurrent theme in all three rounds of the Delphi study and, as reported above, the role of evidence in decommissioning processes was one of only five statements upon which the expert panel could not reach a consensus. This is perhaps unsurprising when we consider that, as with many healthcare decision-making and implementation processes, there is ‘huge looseness, randomness and disconnection … the outcome of which is determined by when, where and how streams of problems, potential solutions, participants and choice opportunities come together and match-up (or not)’ [22]. There are many parallels with the process of how healthcare organizations—at the other end of the innovation continuum—adopt new treatments and services. Key characteristics of these processes have been identified [23] as including: the importance of the history, culture, and quality of interprofessional relationships; the vital role of power and politics in determining the outcome of decision-making processes; the impact of different types of formal and informal decision-making processes (and that a short-term perspective typically predominates); and that professionalism can be a negative influence (*i.e.*, the existence of many different professional groups in healthcare—each with a different perspective, evidence and knowledge base, and skill set—can act as a barrier to implementation). The findings from our Delphi study bear some witness to how very similar processes are influential in the context of implementing decommissioning decisions.

### Relationships and political dimension

There were some additional insights that were more specific to decommissioning, particularly relating to the more political aspects of change as touched on above. They include: the nature of incentives—‘It’s more of a ‘stick’ of legislation and budgets than the ‘carrot’ of long-term quality gain’ (UK, policy, round three); patient and public views—‘I’ve seen plenty of decisions which flew in the face of patient and public views’ (UK, practice, round three), ’patient/public views, expressed loudly, have blocked decommissioning. Stronger in blocking than supporting’ (Australia, research, round three), ‘patient and public views are rarely in this direction’ (UK, policy, round three); and the role of the media—‘the media can be force for good or bad, depending on the quality of communications’ (UK, policy, round two). Nor should we neglect the capital ‘P’ political aspects. Here, several participants highlighted the influential role of nationally and locally elected politicians on the outcomes of decommissioning decision-making processes: ‘politicians have an undue influence over decision making processes … decision makers fear taking decisions which will reflect badly on elected politicians’ (UK, research, round three); ‘local politicians often struggle to think strategically—especially if there is a backlash from constituents and there is an election that year’ (UK, policy/practice, round three); and ‘decommissioning feels inherently political to me.’ (UK, policy, round three). Published case studies of decommissioning processes recount similar experiences of political resistance and policy subversion [24].

Overall , therefore, we would draw a parallel between decommissioning processes and other stories of healthcare policy implementation [22] and suggest that the contemporary ‘state of the art’ of decommissioning is strongly reminiscent of the ‘irrational’ [25,26], and ‘garbage can’ [27] models of decision processes found in the policy and organisation studies literature of the 1970s and 1980s. In particular, the garbage can model was developed to explain the patterns of decision making and innovation in organisations that experience extremely high uncertainty. Cohen, March, and Olson, the originators of the model, called the highly uncertain conditions an ‘organised anarchy’ , suggesting this was caused by three characteristics: problematic preferences (goals, problems, alternatives, and solutions are ill-defined; ambiguity characterises each step of a decision or organisational change process); unclear, poorly understood technology (cause and effect relationships within the organisation are difficult to identify; an explicit database that applies to decisions is not available); and staff turnover (staff are busy and only have limited time to allocate to any one problem or task; participation in any given decision will be fluid and limited). Whilst not of all of these conditions might directly apply to all decommissioning decisions and processes in the contemporary healthcare context, the results (and particularly the open comments) from the Delphi study would suggest that many would be recognized by those with experience of observing or leading such processes. One proposed way forward would be to build on the findings and best practice recommendations in this study and to now co-produce with service users, practitioners, policy makers and researchers a similar set of actions directly relating to informing decommissioning decisions as advocated by Greenhalgh *et al.* [21] for delivering ‘real’ evidence based medicine; significantly, the first of these concerns making ‘the ethical care of the patient [the] top priority.’

### Limitations

There are several limitations to our findings. First, the expertise of the participants in the Delphi study was heavily weighted towards the UK healthcare context as well as to publicly funded, or mixed funding, healthcare systems more generally. Whilst we believe the findings are likely to be broadly generalisable to any publicly funded, or mixed funded, healthcare system they are clearly likely to be less relevant to a fully privately funded system where different environmental and market characteristics might reasonably be assumed to impact on the implementation of decision-making relating to decommissioning. Second, the participants were also weighted towards specific forms of decommissioning by the nature of their experiences and expertise. As outlined in the introduction to this paper there are several different forms of decommissioning and further (either broader consensus-based studies or empirical research) would be required to discern whether these different forms each require different approaches to decision-making and implementation. Third, our responses will inevitably reflect the groups included in the study sample and by extension those excluded. Our attempts to recruit citizens and service users to the Delphi study were unsuccessful, largely because those invited did not recognize themselves as ‘experts’ on the topic. It is noticeable that patient and public engagement did not emerge as a best practice recommendation. We believe that future enquiry should prioritize these hitherto neglected perspectives. Finally, our sample size is insufficiently large to generate meaningful comparisons between the three participant groups represented. However, this might be the focus of future research endeavor; for example, via implementation of a large-scale survey based on the themes identified here.

## Conclusions

The study presented here generates new and timely insights into the factors that shape decommissioning activities in healthcare contexts. Although we identified a strong consensus as to what *should* drive decommissioning—namely considerations of cost-effectiveness, quality and patient safety, and clinical effectiveness—perhaps the most striking finding from the Delphi study is the stark contrast between these ideal drivers and the factors that are most influential in practice. This gap between the rhetoric and reality of decommissioning is at the heart of the decommissioning challenge in healthcare policy and practice; the best practice recommendations identified in this study—which we grouped into three categories: change management and implementation; evidence and information; and relationships and political dimensions—can be seen as contemporary responses or strategies to manage the tensions reflected in our findings.

## Endnote

^a^Williams I, Kimberly J, Robert G, Dickinson H, Mannion R and Harlock J. ‘Slimming down: the challenge of service removal, replacement and reduction in healthcare.’ Manuscript submitted for publication
